# Non-contiguous finished genome sequence and description of *Clostridium senegalense* sp. nov.

**DOI:** 10.4056/sigs.2766062

**Published:** 2012-07-20

**Authors:** Ajay Kumar Mishra, Jean-Christophe Lagier, Catherine Robert, Didier Raoult, Pierre-Edouard Fournier

**Affiliations:** 1 Unité de Recherche sur les Maladies Infectieuses et Tropicales Emergentes, UMR CNRS, Faculté de médecine, Aix-Marseille Université

**Keywords:** *Clostridium senegalense*, genome

## Abstract

*Clostridium senegalense* strain JC122^T^, is the type strain of *Clostridium senegalense* sp. nov., a new species within the genus *Clostridium*. This strain, whose genome is described here, was isolated from the fecal flora of a healthy patient. *C. senegalense* strain JC122^T^ is an obligate anaerobic Gram-positive rod-shaped bacterium. Here we describe the features of this organism, together with the complete genome sequence and annotation. The 3,893,008 bp long genome (1 chromosome but no plasmid) exhibits a G+C content of 26.8% and contains 3,704 protein-coding and 57 RNA genes, including 6 rRNA genes.

## Introduction

*Clostridium senegalense* strain JC122^T^ (= CSUR P152 = DSM 25507), is the type strain of *Clostridium senegalense* sp. nov. This bacterium is a Gram-positive, anaerobic, spore-forming, indole negative rod-shaped bacterium that was isolated from the stool of a healthy Senegalese patient as part of a “culturomics” study aiming at cultivating individually all species within human feces.

Since 1995 and the sequencing of the first bacterial genome, that of *Haemophilus influenzae*, more than 2,000 bacterial genomes have been sequenced [1]. This was permitted by technical improvements as well as increased interest in having access to the complete genetic information encoded by bacteria. In the same time, biological tools for the definition of new bacterial species have not evolved, DNA-DNA hybridization still being considered as the gold standard [2] despite its drawbacks and the taxonomic revolution that has resulted from the comparison of 16S rDNA sequences [3]. In this manuscript, we propose to use genomic data, in addition to phenotypic information [4], to describe a new *Clostridium* species.

Here we present a summary classification and a set of features for *C. senegalense* sp. nov. strain JC122^T^ (= CSUR P152= DSM 25507) together with the description of the complete genomic sequencing and annotation. These characteristics support the circumscription of the species *C. senegalense*.

The genus *Clostridium* (Prazmowski, 1880) was created in 1880 [5] and consists of obligate anaerobic rod-shaped bacilli capable of producing endospores [5]. More than 180 species have been described to date. Members of the genus *Clostridium* are mostly environmental bacteria or associated with the commensal digestive flora of mammals. However, several are major human pathogens, including *C. botulinum*, *C. difficile* and *C. tetani* [6,7]. Few species, such as *C. butyricum* and *C. pasteurianum*, fix nitrogen and have gained importance in agricultural and industrial applications [8,9].

### Classification and features

**Figure 1:**
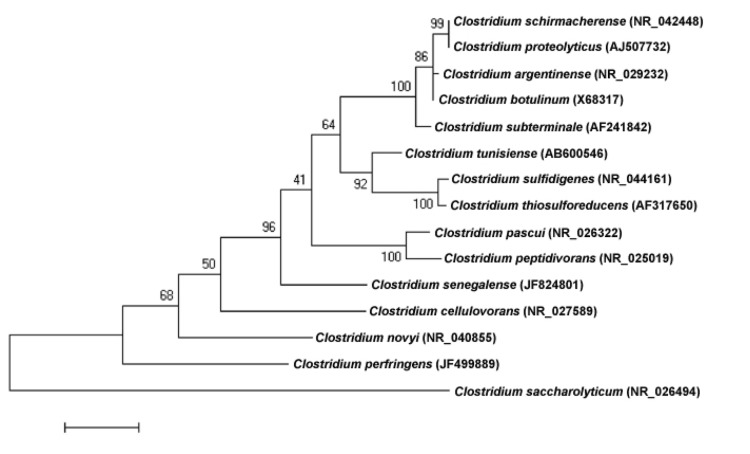
Phylogenetic tree highlighting the position of *Clostridium senegalense* strain JC122^T^ relative to other type strains within the *Clostridium* genus. GenBank accession numbers are indicated in parentheses. Sequences were aligned using CLUSTALW, and phylogenetic inferences obtained using the maximum-likelihood method within the MEGA software. Numbers at the nodes are bootstrap values obtained by repeating the analysis 500 times to generate a majority consensus tree. *Clostridium saccharolyticum* was used as an outgroup. The scale bar represents a 2% nucleotide sequence divergence.

A stool sample was collected from a healthy 16-year-old male Senegalese volunteer patient living in Dielmo (a rural village in the Guinean-Sudanian zone in Senegal), who was included in a research protocol. The patient gave an informed and signed consent, and the agreement of the National Ethics Committee of Senegal and the local ethics committee of the IFR48 (Marseille, France) were obtained under agreements 09-022 and 11-017. The fecal specimen was preserved at -80°C after collection and sent to Marseille. Strain JC122 (Table 1) was isolated in June 2011 by anaerobic cultivation on 5% sheep blood-enriched Columbia agar (BioMerieux, Marcy l’Etoile, France). This strain exhibited a 95.6% nucleotide sequence similarity with *C. subterminale* [22], and occupied an intermediate phylogenetic position between *C. cellulovorans* and *C. peptidivorans* (Figure 1). Although sequence similarity of the 16S operon is not uniform across taxa, this value was lower than the 98.7% 16S rRNA gene sequence threshold recommended by Stackebrandt and Ebers to delineate a new species without carrying out DNA-DNA hybridization [23].

**Table 1:** Classification and general features of *Clostridium senegalense* strain JC122^T^

**MIGS ID**	**Property**	**Term**	**Evidence code^a^**
	Current classification	Domain *Bacteria*	TAS [10]
		Phylum *Firmicutes*	TAS [11-13]
		Class *Clostridia*	TAS [14,15]
		Order *Clostridiales*	TAS [16,17]
		Family *Clostridiaceae*	TAS [16,18]
		Genus *Clostridium*	TAS [16,19,20]
		Species *Clostridium senegalense*	IDA
		Type strain JC122^T^	IDA
	Gram stain	positive	IDA
	Cell shape	rod-shaped	IDA
	Motility	motile	IDA
	Sporulation	sporulating	IDA
	Temperature range	mesophilic	IDA
	Optimum temperature	37°C	IDA
MIGS-6.3	Salinity	growth in BHI medium + 5% NaCl	IDA
MIGS-22	Oxygen requirement	anaerobic	IDA
	Carbon source	unknown	NAS
	Energy source	unknown	NAS
MIGS-6	Habitat	human gut	IDA
MIGS-15	Biotic relationship	free living	IDA
MIGS-14	Pathogenicity	unknown	NAS
	Biosafety level	2	
	Isolation	human feces	
MIGS-4	Geographic location	Senegal	IDA
MIGS-5	Sample collection time	September 2010	IDA
MIGS-4.1	Latitude	13.7167	IDA
MIGS-4.1	Longitude	- 16.4167	IDA
MIGS-4.3	Depth	surface	IDA
MIGS-4.4	Altitude	51 m above sea level	IDA

**^a^**Evidence codes - IDA: Inferred from Direct Assay; TAS: Traceable Author Statement (i.e., a direct report exists in the literature); NAS: Non-traceable Author Statement (i.e., not directly observed for the living, isolated sample, but based on a generally accepted property for the species, or anecdotal evidence). These evidence codes are from the Gene Ontology project [21]. If the evidence is IDA, then the property was directly observed for a live isolate by one of the authors or an expert mentioned in the acknowledgements.

**Figure 2:**
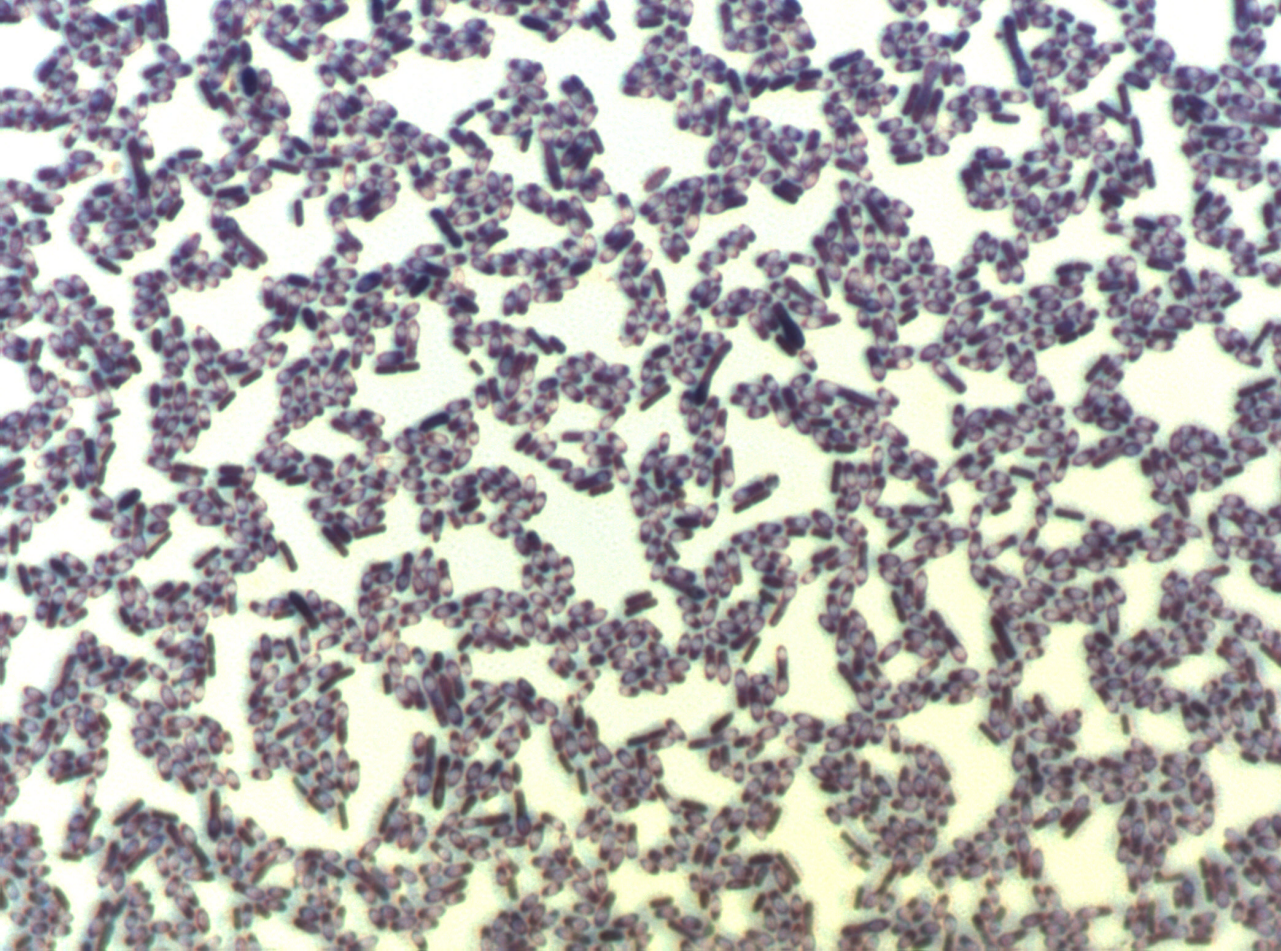
Gram staining of *C. senegalense* strain JC122^T^

**Figure 3:**
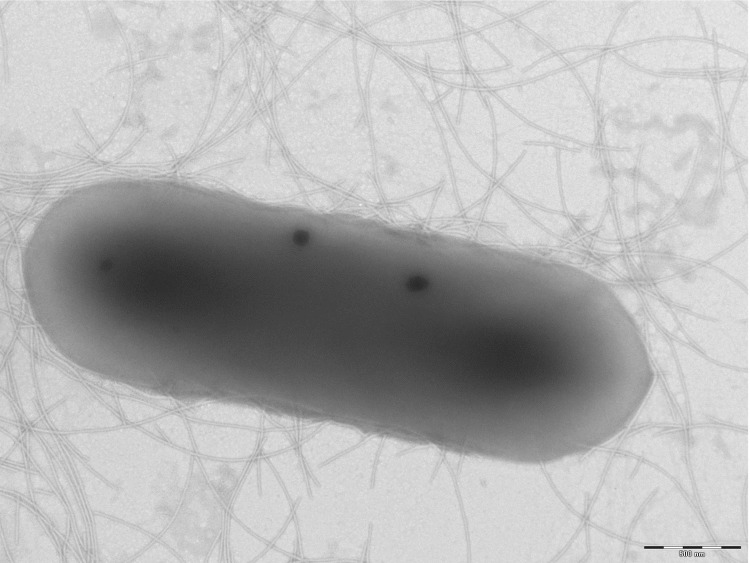
Transmission electron microscopy of *C. senegalense* strain JC122^T^, using a Morgani 268D (Philips) at an operating voltage of 60kV. The scale bar represents 900 nm.

Different growth temperatures (25, 30, 37, 45°C) were tested; no growth occurred at 45°C, growth occurred at 25° and 30°C, and optimal growth was observed at 37°C. Colonies were 2 mm in diameter on blood-enriched Columbia agar and Brain Heart Infusion (BHI) agar. Growth of the strain was tested under anaerobic and microaerophilic conditions using GENbag anaer and GENbag microaer systems, respectively (BioMérieux), and in the presence of air, with or without 5% CO_2_. Growth was achieved only anaerobically. Gram staining showed rod-shaped Gram-positive bacilli able to form spores (Figure 2). The motility test was positive. Cells grown on agar have a mean diameter of 1.1 µm (Figure 3).

Strain JC122^T^ exhibited neither catalase nor oxidase activities. Using API Rapid ID 32A, a positive reaction was observed for arginine dihydrolase, N-acetyl-β-glucosanimidase and pyroglutamic acid arylamidase. Negative reactions were observed for urease, indole and nitrate reduction. *C. senegalense* is susceptible to amoxicillin, imipenem, metronidazole, rifampicin and vancomycin but resistant to trimethoprim/sulfamethoxazole.

**Figure 4:**
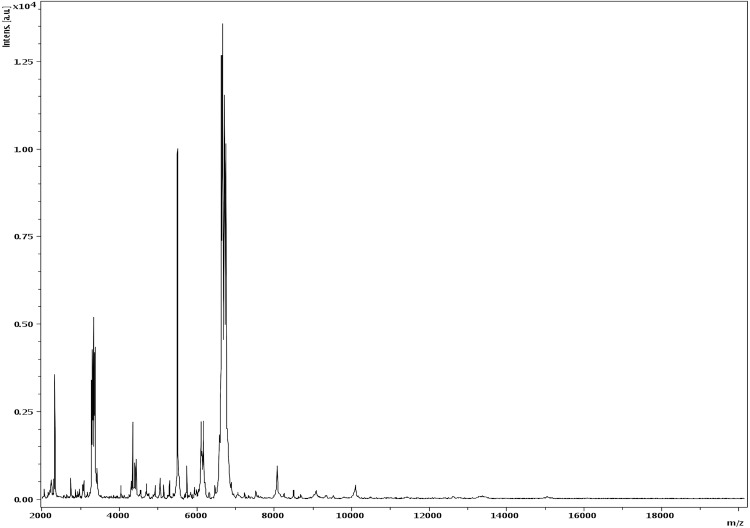
Reference mass spectrum from *C. senegalense* strain JC122^T^. Spectra from 12 individual colonies were compared and a reference spectrum was generated.

Matrix-assisted laser-desorption/ionization time-of-flight (MALDI-TOF) MS protein analysis was carried out as previously described [24]. Briefly, a pipette tip was used to pick one isolated bacterial colony from a culture agar plate and spread it as a thin film on a MTP 384 MALDI-TOF target plate (Bruker Daltonics, Germany). Twelve distinct deposits were done for strain JC122^T^ from twelve isolated colonies. Each smear was overlaid with 2µL of matrix solution (saturated solution of alpha-cyano-4-hydroxycinnamic acid) in 50% acetonitrile, 2.5% tri-fluoracetic acid, and allowed to dry for five minutes. Measurements were performed with a Microflex spectrometer (Bruker). Spectra were recorded in the positive linear mode for the mass range of 2,000 to 20,000 Da (parameter settings: ion source 1 (ISI), 20kV; IS2, 18.5 kV; lens, 7 kV). A spectrum was obtained after 675 shots at a variable laser power. The time of acquisition was between 30 seconds and 1 minute per spot. The twelve JC122^T^ spectra were imported into the MALDI Bio Typer software (version 2.0, Bruker) and analyzed by standard pattern matching (with default parameter settings) against the main spectra of 3,769 bacteria, including spectra from 59 validated *Clostridium* species used as reference data, in the Bio Typer database (updated March 15^th^, 2012). The method of identification includes the m/z from 3,000 to 15,000 Da. For every spectrum, 100 peaks at most were taken into account and compared with the spectra in database. A score enabled the presumptive identification and discrimination of the tested species: a score ≥ 2 with a validated species enabled the identification at the species level; a score ≥ 1.7 but < 2 enabled the identification at the genus level; and a score < 1.7 did not enable any identification. For strain JC122^T^, the obtained score was 1.3, thus suggesting that our isolate was not a member of a known species. We incremented our database with the spectrum from strain JC122^T^ (Figure 4).

## Genome sequencing information

### Genome project history

The organism was selected for sequencing on the basis of its phylogenetic position and 16S rRNA similarity to other members of the genus *Clostridium*, and is part of a “culturomics” study of the human digestive flora aiming at isolating all bacterial species within human feces. It was the 74th genome of a *Clostridium* species and the first genome of *Clostridium senegalense* sp. nov. The Genbank accession number is CAEV00000000 and consists of 191 contigs. Table 2 shows the project information and its association with MIGS version 2.0 compliance.

**Table 2:** Project information

**MIGS ID**	**Property**	**Term**
MIGS-31	Finishing quality	High-quality draft
MIGS-28	Libraries used	One 454 paired end 3-kb library
MIGS-29	Sequencing platforms	454 GS FLX Titanium
MIGS-31.2	Sequencing	35×
MIGS-30	Assemblers	Newbler version 2.5.3
MIGS-32	Gene calling method	Prodigal
	INSDC ID	109297
	Genbank ID	CAEV00000000
	Genbank Date of Release	July 25, 2011
	Gold ID	Gi13536
MIGS-13	Project relevance	Study of the human gut microbiome

### Growth conditions and DNA isolation

*C. senegalense* sp. nov. strain JC122^T^, CSUR P152 = DSM 25507, was grown on blood agar medium at 37°C. Five petri dishes were spread and resuspended in 5x100µl of G2 buffer (EZ1 DNA Tissue kit, Qiagen). A first mechanical lysis was performed by glass powder on the Fastprep-24 device (Sample Preparation system) from MP Biomedicals, USA) using 2x20 seconds cycles. DNA was then treated with 2.5 µg/µL lysozyme (30 minutes at 37°C) and extracted through the BioRobot EZ 1 Advanced XL (Qiagen). The DNA was then concentrated and purified on a Qiamp kit (Qiagen). The yield and the concentration was measured by the Quant-it Picogreen kit (Invitrogen) on a Genios_Tecan fluorometer at 70.7 ng/µl.

### Genome sequencing and assembly

This project was loaded twice on a 1/4 region for the paired end application and once on a 1/8 region for the shotgun on PTP Picotiterplates. The shotgun library was constructed with 500ng of DNA as described by the manufacturer Roche with the GS Rapid library Prep kit. For the paired-end sequencing, DNA (5µg) was mechanically fragmented on the Hydroshear device (Digilab, Holliston, MA, USA) with an enrichment size of 3-4kb. The DNA fragmentation was visualized using an Agilent 2100 BioAnalyzer on a DNA labchip 7500 to yield an optimal size of 3.6 kb. The library was constructed according to the 454_Titanium paired end protocol and manufacturer. Circularization and nebulization were performed and generated a pattern with an optimum at 561 bp. After PCR amplification through 15 cycles followed by double size selection, the single stranded paired end library was then quantified on the Quant-it Ribogreen kit (Invitrogen) on the Genios_Tecan fluorometer at 52pg/µL. The library concentration equivalence was calculated as 1.7E+08 molecules/µL. The library was held at -20°C until use.

The shotgun library was clonally amplified with 3cpb in 3 emPCR reactions and the paired end library was amplified with lower cpb (1cpb) in 4 emPCR reactions with the GS Titanium SV emPCR Kit (Lib-L) v2. The yield of the emPCR was 5.37% for the shotgun and 19.27% for the paired end according to the quality expected by the range of 5 to 20% from the Roche procedure. A total of 340,000 beads for the 1/8 region for the shotgun and 790,000 beads on the 1/4 region for the paired end were loaded on the GS Titanium PicoTiterPlates (PTP Kit 70×75) and sequenced with the GS Titanium Sequencing Kit XLR70.

The runs were performed overnight and then analyzed on the cluster through the gsRunBrowser and gsAssembler_Roche. The global 383,079 passed filter sequences generated 96.50 Mb with a length average of 277bp. These sequences were assembled using the Newbler software from Roche with 90% identity and 40 bp as overlap. Fourteen scaffolds and 120 large contigs (>1500bp) were obtained, for a genome size of 3,893,008 bp.

### Genome annotation

Open Reading Frames (ORFs) were predicted using Prodigal [25] with default parameters but the predicted ORFs were excluded if they were spanning a sequencing gap region. The predicted bacterial protein sequences were searched against the GenBank database [26] and the Clusters of Orthologous Groups (COG) database using BLASTP. The tRNAScanSE tool [27] was used to find tRNA genes, whereas ribosomal RNAs were found by using RNAmmer [28] and BLASTn against the GenBank database. ORFans were identified if their BLASTP *E*-value was lower than 1e-03 for alignment length greater than 80 amino acids. If alignment lengths were smaller than 80 amino acids, we used an *E*-value of 1e-05. Such parameter thresholds have already been used in previous works to define ORFans.

To estimate the mean level of nucleotide sequence similarity at the genome level between *Clostridium* species, we compared the ORFs only using BLASTN and the following parameters: a query coverage of ≥ 70% and a minimum nucleotide length of 100 bp.

## Genome properties

**Figure 5:**
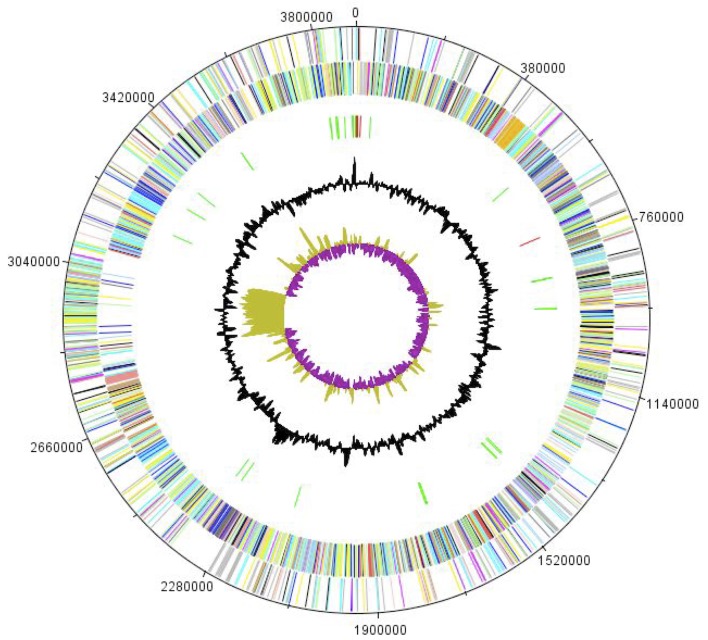
Graphical circular map of the chromosome. From outside to the center: Genes on the forward strand (colored by COG categories), genes on the reverse strand (colored by COG categories), RNA genes (tRNAs green, rRNAs red), GC content, and GC skew.

The genome of *C. senegalense* sp. nov. strain JC122^T^ is 3,893,008 bp long (1 chromosome, but no plasmid) with a 26.8% G + C content of (Figure 5 and Table 3). Of the 3,761 predicted genes, 3,704 were protein-coding genes, and 57 were RNAs. Six rRNA genes (one 16S rRNA, one 23S rRNA and four 5S rRNA) and 51 predicted tRNA genes were identified in the genome. A total of 2,560 genes (68.06%) were assigned a putative function. Four hundred forty-three genes were identified as ORFans (12%). The remaining genes were annotated as hypothetical proteins. The properties and the statistics of the genome are summarized in Table 3.The distribution of genes into COGs functional categories is presented in Table 4.

**Table 3:** Nucleotide content and gene count levels of the genome

**Attribute**	**Value**	% of total^a^
Genome size (bp)	3,893,008	100
DNA coding region (bp)	3,126,069	80.30
DNA G+C content (bp)	1,043,326	26.8
Total genes	3,761	100
RNA genes	57	1.51
Protein-coding genes	3,704	98.48
Genes with function prediction	2,677	71.17
Genes assigned to COGs	2,560	68.06
Genes with peptide signals	169	4.49
Genes with transmembrane helices	973	25.87

^a^ The total is based on either the size of the genome in base pairs or the total number of protein coding genes in the annotated genome

**Table 4:** Number of genes associated with the 25 general COG functional categories

**Code**	**Value**	**% age**^a^	**Description**
J	183	4.94	Translation, ribosomal structure and biogenesis
A	0	0	RNA processing and modification
K	260	7.02	Transcription
L	165	4.45	Replication, recombination and repair
B	1	0.03	Chromatin structure and dynamics
D	28	0.75	Cell cycle control, mitosis and meiosis
Y	0	0	Nuclear structure
V	155	4.18	Defense mechanisms
T	202	5.45	Signal transduction mechanisms
M	134	3.62	Cell wall/membrane biogenesis
N	70	1.88	Cell motility
Z	0	0	Cytoskeleton
W	0	0	Extracellular structures
U	37	0.99	Intracellular trafficking and secretion
O	74	1.99	Posttranslational modification, protein turnover, chaperones
C	170	4.59	Energy production and conversion
G	102	2.75	Carbohydrate transport and metabolism
E	226	6.10	Amino acid transport and metabolism
F	79	2.13	Nucleotide transport and metabolism
H	104	2.80	Coenzyme transport and metabolism
I	64	1.73	Lipid transport and metabolism
P	136	3.67	Inorganic ion transport and metabolism
Q	61	1.65	Secondary metabolites biosynthesis, transport and catabolism
R	426	11.50	General function prediction only
S	232	6.26	Function unknown
-	1,198	32.34	Not in COGs

^a^ The total is based on the total number of protein coding genes in the annotated genome.

## Comparison with the genomes from other *Clostridium* species

Seventy-three genomes are currently available for *Clostridium* species. Here, we compared the genome sequence of *C. senegalense* strain JC122^T^ with those of *C. botulinum* strain ATCC 19397 and *C. cellulovorans* strain, ATCC 35296.

The draft genome sequence of *C. senegalense* strain JC122^T^ has a similar size to that of *C. botulinum* (3.89 and 3.94 Mb, respectively), but a smaller size than *C. cellulovorans* (5.2 Mb). The G+C content of *C. senegalense* was lower than *C. botulinum* and *C. cellulovorans* (26.8% vs 28.2 and 31.2%, respectively). The gene content of *C. senegalense* is comparable to that of *C. botulinum* (3,761 and 3,750, respectively) but is smaller to that of *C. cellulovorans* (4,500). The ratios of genes per Mb and numbers of genes assigned to COGs of *C. senegalense* and *C. botulinum* are similar (974 *vs* 946 and 2,560 vs 2,549, respectively), but larger than the ratio of genes per Mb (844) and smaller than the number of genes assigned to COGs of *C. cellulovorans* (2,927). However, the distribution of genes into COG categories (Table 4) was similar in all the three compared genomes.

In addition, *C. senegalense* shared a mean 84.9% (range 77.4-95%) and 82.79% (range 77.2-92.3%) sequence similarity with *C. botulinum* and *C. cellulovorans* respectively at the genome level.

On the basis of phenotypic, phylogenetic and genomic analyses, we formally propose the creation of *Clostridium senegalense* sp. nov. which contains the strain JC122^T^. This bacterium has been found in Senegal.

### Description of *Clostridium senegalense* sp. nov.

*Clostridium senegalense* (se.ne.gal.e′n.sis. L. gen. masc. n. *senegalensis*, pertaining to, or originating from Senegal, the country from which the specimen was isolated). 

Colonies are 2 mm in diameter on blood-enriched Columbia agar and Brain Heart Infusion (BHI) agar. Cells are rod-shaped with a mean diameter of 1.1 μm. Optimal growth is achieved anaerobically. No growth is observed in aerobic conditions. Growth occurs between 25-37°C, with optimal growth observed at 37°C, in BHI medium + 5% NaCl. Cells stain Gram-positive, are endospore-forming, and motile. Catalase, oxidase, urease, indole and nitrate reduction activity are absent. Arginine dihydrolase, N-acetyl-β-glucosanimidase and pyroglutamic acid arylamidase activity are present. Cells are susceptible to amoxicillin, imipenem, metronidazole, rifampicin and vancomycin but resistant to trimethoprim/sulfamethoxazole. The G+C content of the genome is 26.8%. 

The type strain is JC122^T^ (= CSUR P152 = DSM 25507) was isolated from the fecal flora of a healthy patient in Senegal.
